# A Heterogeneous Multi-Output Stacked Learning Framework for Mechanical Property Prediction of FDM-Printed ASA: Experimental Validation

**DOI:** 10.3390/polym18172100

**Published:** 2026-08-29

**Authors:** Afnan Haider Khan, Farheen Umar, Umar Ayoub, Mushaf Ur Rehman Khan, Shahbaz Haneef, Muhammad Farooq Siddique

**Affiliations:** 1 Department of Mechanical Engineering, University of Engineering and Technology, Mardan, Charsadda Road, Mardan 23200, Khyber Pakhtunkhwa, Pakistan; afnan@uetmardan.edu.pk (A.H.K.); engr.mushaf@uetmardan.edu.pk (M.U.R.K.); 2 Department of Physics, University of Peshawar, University Road, Peshawar 25000, Khyber Pakhtunkhwa, Pakistan; farheenumarf@uop.edu.pk; 3 Department of Computer Science, University of Engineering and Technology, Mardan, Charsadda Road, Mardan 23200, Khyber Pakhtunkhwa, Pakistan; uayoub181003@gmail.com; 4 Department of Physical and Numerical Sciences, Abdul Wali Khan University, Mardan 23200, Khyber Pakhtunkhwa, Pakistan; baz.physics@gmail.com

**Keywords:** polymer additive manufacturing, fused deposition modelling (FDM), acrylonitrile styrene acrylate (ASA), machine learning, heterogeneous stacked ensemble learning, multi-output prediction

## Abstract

Accurate prediction of the mechanical performance of polymer components fabricated by fused deposition modelling (FDM) remains challenging owing to the complex nonlinear relationships between process parameters and material properties, limiting reliable process planning and broader industrial adoption of polymer additive manufacturing. This study develops and experimentally validates a heterogeneous multi-output stacked ensemble learning framework for the simultaneous prediction of tensile strength, flexural strength, compressive strength, Rockwell hardness, and Charpy impact strength of acrylonitrile styrene acrylate (ASA), a high-performance engineering thermoplastic with excellent weatherability and ultraviolet resistance that remains comparatively underexplored in data-driven FDM research. A Definitive Screening Design (DSD) was employed to investigate eight critical process parameters: extrusion temperature (ET), bed temperature (BT), infill density (ID), layer height (LH), print speed (PS), raster angle (RA), build orientation (BO), and cooling fan speed (CFS). Multiple supervised learning algorithms were systematically benchmarked, and the highest-performing complementary models were integrated into a heterogeneous stacked ensemble for simultaneous multi-output prediction. The proposed framework achieved an overall R^2^ of 0.9943 with an overall RMSE of 0.9758, while the individual prediction models attained R^2^ values ranging from 0.9898 to 0.9967. Beyond improving predictive accuracy, the proposed AI-assisted framework provides a data-driven basis for mechanical-property prediction and establishes a surrogate modelling framework that may subsequently be coupled with dedicated optimization or decision-making methods.

## 1. Introduction

Reliable predictive modelling of manufacturing-induced material performance has become a cornerstone of intelligent manufacturing, where accurate characterization of complex process–property relationships is essential for reducing experimental effort, accelerating product development, and enabling data-driven decision-making [1,2]. Among advanced manufacturing technologies, fused deposition modelling (FDM) has matured from a rapid prototyping tool into a viable manufacturing process for producing functional polymer components across aerospace, automotive, biomedical, construction, and consumer sectors owing to its design flexibility, cost-effectiveness, and material versatility [3,4,5].

Despite these advantages, accurately predicting the mechanical performance of FDM-printed components remains a significant challenge. Multiple processing parameters interact through highly nonlinear process–structure–property relationships that govern interlayer bonding, porosity, dimensional accuracy, residual stresses, and material anisotropy, resulting in substantial variations in tensile, flexural, compressive, hardness, and impact properties even under nominally similar processing conditions [6,7,8]. Although extensive experimental investigations have improved the understanding of individual process parameters, exhaustive experimentation across a high-dimensional and strongly coupled design space remains expensive, time-consuming, and often impractical. Therefore, data-driven predictive modelling has become indispensable for intelligent additive manufacturing, enabling efficient process optimization while reducing reliance on costly trial-and-error experimentation [9,10].

Conventional statistical analyses, empirical regression models, and design-of-experiments (DOE)-based methodologies have long been employed to investigate the influence of FDM process parameters on mechanical performance. However, these approaches typically rely on predefined mathematical relationships and exhibit limited capability in representing the highly nonlinear, multidimensional, and strongly coupled nature of FDM systems [11,12,13]. Unlike conventional modelling approaches, supervised machine learning (ML) provides a fundamentally different paradigm by learning complex input–output relationships directly from experimental data without requiring explicit assumptions regarding the underlying physical mechanisms [14,15]. Its capability to capture nonlinear feature interactions, identify hidden patterns, and generalize across unseen data has established ML as a key enabling technology for intelligent manufacturing, digital twins, predictive analytics, and data-driven process optimization. Beyond improving predictive accuracy, ML also facilitates knowledge extraction from experimental datasets, providing valuable insights into the interactions between manufacturing parameters and engineering responses while supporting informed process optimization and material qualification [16,17].

Recent years have witnessed rapid growth in the application of supervised machine learning for predictive modelling and process optimization in fused deposition modelling, driven by the need to reduce experimental effort while accurately capturing the complex nonlinear relationships between processing parameters and engineering performance. Early studies primarily focused on identifying the most suitable learning algorithms for predicting individual mechanical responses [18,19,20]. For example, Veeman et al. [21] evaluated multiple ML algorithms for predicting the hardness of FDM-fabricated ABS components and identified Random Forest (RF) as the best-performing model (R^2^ = 0.9136). A more comprehensive benchmark was subsequently reported by Özkül et al. [22], who evaluated 25 supervised learning algorithms for ABS components, demonstrating that the Multilayer Perceptron (MLP) achieved the highest prediction accuracy (R^2^ > 0.99) for tensile and flexural strength. As predictive capability matured, research shifted from property prediction towards intelligent process optimization, where Borah and Chandrasekaran [23] integrated Artificial Neural Networks (ANNs) with Teaching–Learning-Based Optimization (TLBO) and Non-dominated Sorting Genetic Algorithm II (NSGA-II) to simultaneously predict and optimize the mechanical performance of FDM-printed PEEK.

Subsequent research increasingly emphasized prediction robustness, optimization efficiency, and model interpretability. Pazarcıkcı et al. [24] demonstrated the superior performance of Random Forest for predicting the tensile, flexural, and impact properties of PC-ABS (R^2^ = 0.94–0.99), while Shen et al. [25] combined Particle Swarm Optimization (PSO) and SHAP with multiple ML models to enhance prediction accuracy and explain the influence of processing parameters on printed-line characteristics. Extending these predictive modelling efforts to engineering-grade polymers, Bolat et al. [26] developed machine learning and deep learning models for predicting the hardness and surface roughness of ASA under varying printing and post-processing conditions, demonstrating the superior predictive capability of a hybrid SVR–1D CNN framework. More recently, research has expanded beyond single-response prediction to multi-response learning, where multiple responses are modelled simultaneously within a unified framework. By exploiting shared information among related prediction tasks, these approaches can improve learning efficiency and predictive generalization while reducing the need for separate models [27,28]. Naidu et al. [29] proposed a Multi-Target Machine Learning (MTML) framework for simultaneously predicting multiple mechanical properties of PLA-, ABS-, and PETG-based components, whereas Abu-Shams et al. [30] demonstrated the effectiveness of Ensemble Regression Trees (ERTs) for jointly modelling tensile strength and energy consumption in material extrusion. Extending these developments toward intelligent decision-making, Chowdhury et al. [31] integrated machine learning surrogate models with a hybrid NSGA-II–TOPSIS optimization framework to balance weld strength and processing time in FDM, illustrating the growing role of machine learning in predictive optimization of additive manufacturing processes.

Nevertheless, several important challenges remain. Existing studies have predominantly focused on conventional polymers such as PLA, ABS, PETG, PEEK, and PC-ABS, whereas acrylonitrile styrene acrylate (ASA) has received comparatively little attention despite its superior weatherability, ultraviolet resistance, thermal stability, and long-term durability for engineering applications. Furthermore, although recent studies have demonstrated the potential of ensemble learning and multi-target prediction, these approaches largely rely on single predictive models or homogeneous ensemble architectures, limiting their ability to exploit the complementary strengths of diverse learning algorithms. As a result, a unified and experimentally validated heterogeneous stacked learning framework for the simultaneous prediction of multiple mechanical properties of FDM-fabricated ASA components has yet to be established.

Building upon these advances while addressing the identified limitations, this study develops and experimentally validates a heterogeneous multi-output stacked machine learning framework for predicting the mechanical performance of FDM-fabricated ASA components. A comprehensive Definitive Screening Design (DSD)-based experimental dataset comprising eight critical FDM process parameters and five ASTM-characterized mechanical properties was used to develop and validate the proposed framework. Multiple supervised ML algorithms were systematically benchmarked to identify the highest-performing heterogeneous learning models. The two best-performing heterogeneous models, Support Vector Regression (SVR) and Artificial Neural Network (ANN), were subsequently integrated using Ridge Regression (RR) as the meta-learner within a stacked learning architecture, enabling the combination of their complementary predictive characteristics for simultaneous multi-output prediction. The resulting framework establishes a unified and experimentally validated predictive model for simultaneous prediction of five mechanical properties of engineering-grade ASA, providing a data-driven foundation for intelligent process optimization and machine learning-assisted additive manufacturing.

The scope of the present study is focused on predictive modelling and experimental validation rather than explicit process optimization or parameter-importance analysis. Accordingly, no optimum FDM parameter combination is claimed in this work. Instead, the experimentally validated predictive models are developed as surrogate tools that can subsequently be coupled with optimization or decision-making algorithms to identify desirable processing conditions within the investigated parameter space.

## 2. Materials and Methods

### 2.1. Material, Experimental Design, and Dataset Generation

**Figure 1:**
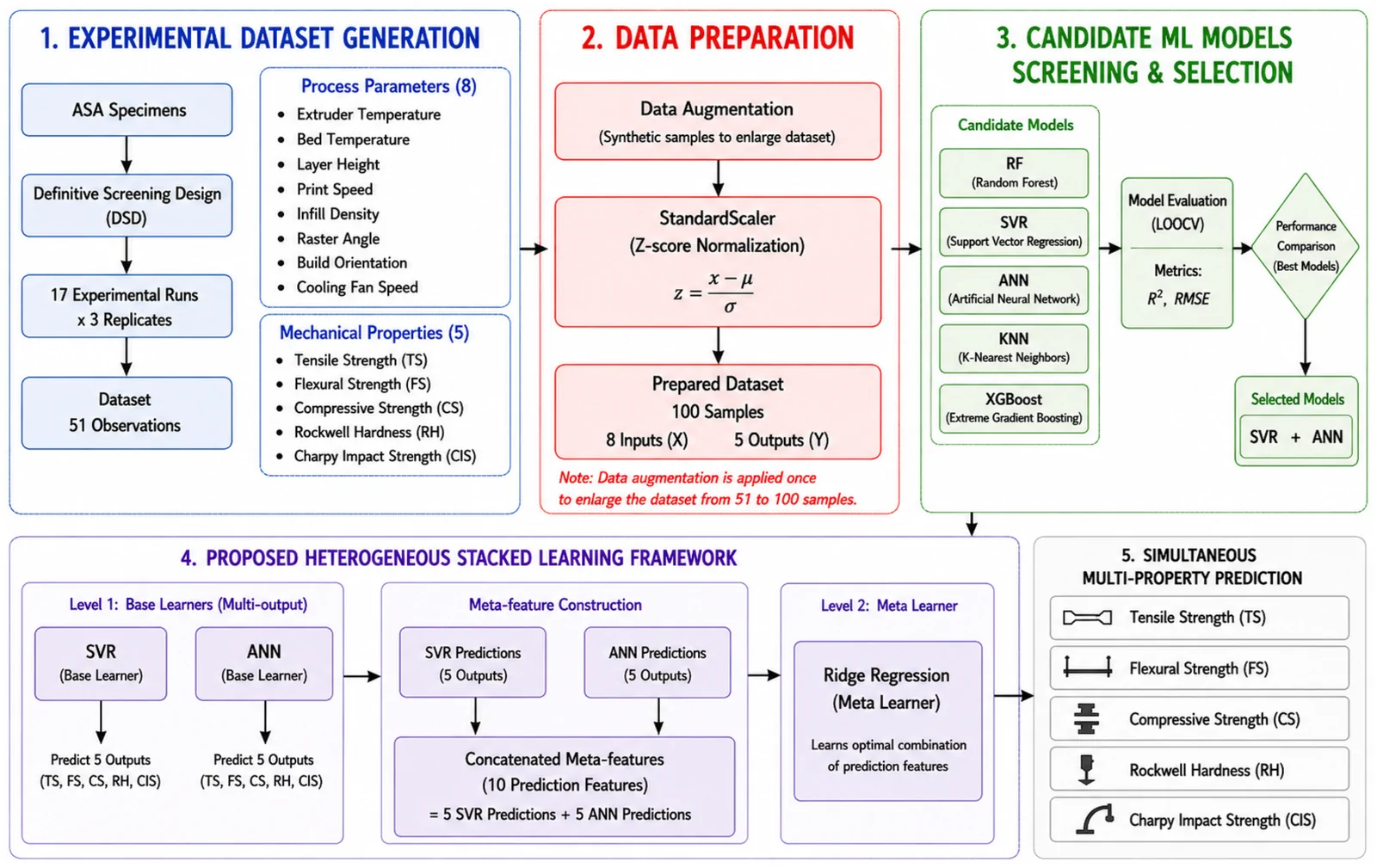
Workflow of the proposed heterogeneous multi-output stacked learning framework for simultaneous multi-property prediction.

The experimental dataset used to develop and validate the proposed heterogeneous multi-output stacked learning framework was generated through fused deposition modelling (FDM) of acrylonitrile styrene acrylate (ASA) specimens. Commercial ASA filament (ERYONE, Shenzhen, China) with a nominal diameter of 1.75 mm was employed owing to its excellent weatherability, thermal stability, and mechanical performance, making it well suited for engineering applications. All specimens were fabricated using a QIDI FDM printer (QIDI Technology, Shaoxing, China) equipped with a 0.4 mm nozzle. The overall workflow adopted in this study is illustrated in Figure 1.

Eight FDM process parameters, namely extruder temperature (ET), bed temperature (BT), infill density (ID), layer height (LH), print speed (PS), raster angle (RA), build orientation (BO), and cooling fan speed (CFS), were selected as predictor variables. Their corresponding levels are summarized in Table 1. To systematically investigate the effects of these variables while maintaining experimental efficiency, a Definitive Screening Design (DSD) was employed. The experimental design comprised 17 unique processing conditions, each fabricated in triplicate, resulting in a total of 51 experimental observations. The corresponding DSD matrix is presented in Table 2.

**Figure 2:**
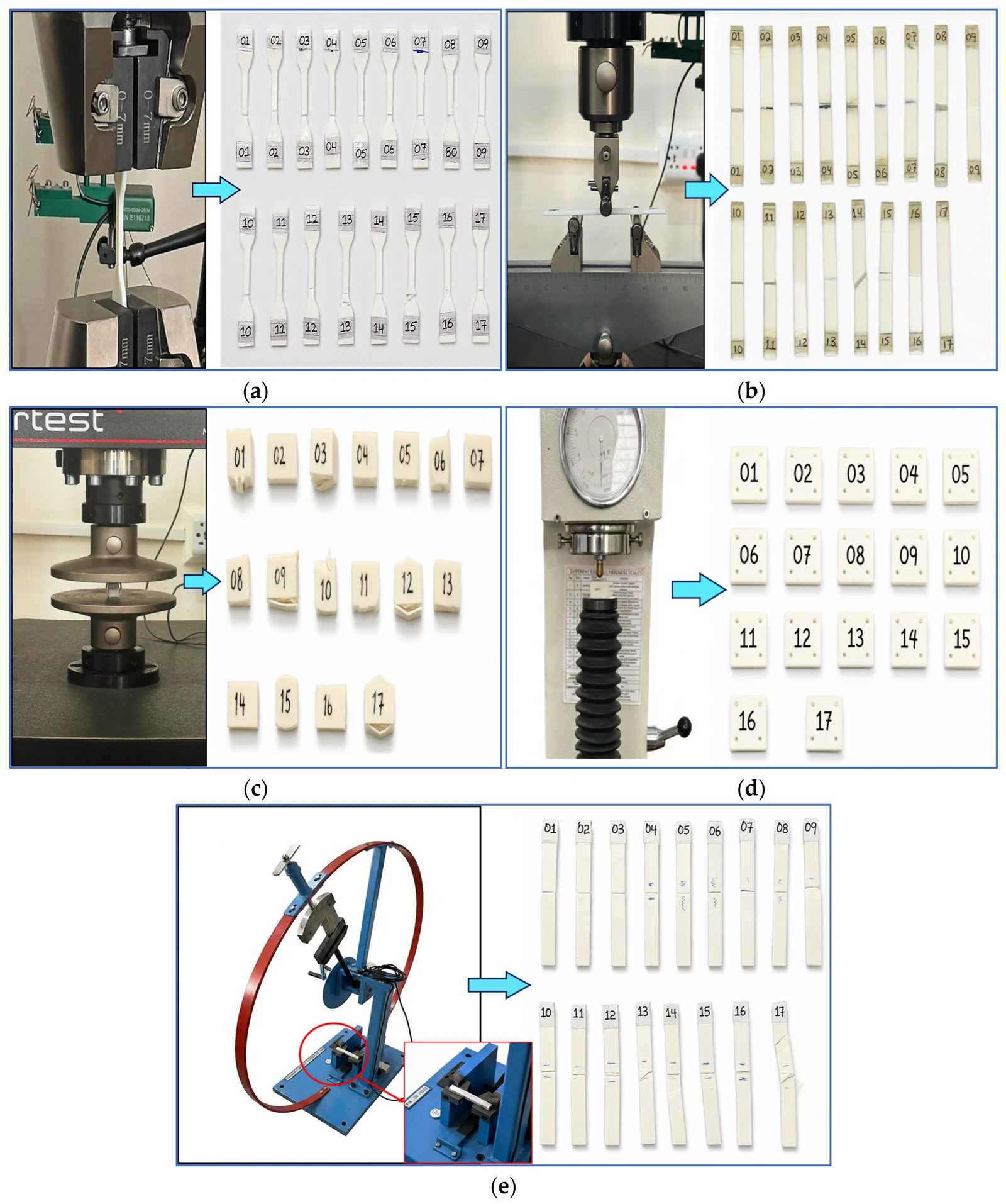
Experimental workflow showing the mechanical testing procedures and representative tested specimens: (**a**) tensile, (**b**) flexural, (**c**) Rockwell hardness, (**d**) Charpy impact, and (**e**) compression tests.

The mechanical performance of the fabricated specimens was evaluated through tensile strength (TS), flexural strength (FS), compressive strength (CS), Rockwell hardness (RH), and Charpy impact strength (CIS) in accordance with the relevant ASTM standards. The experimental testing procedures together with representative fractured specimens are presented in Figure 2. These five response variables, together with the eight FDM process parameters, constituted the experimental dataset used for subsequent data preprocessing and machine learning (ML) model development.

### 2.2. Data Preprocessing and Candidate Model Screening

The experimental dataset comprised 51 observations generated from 17 unique Definitive Screening Design (DSD) process combinations, each fabricated and mechanically tested in triplicate. Although the DSD provided efficient coverage of the eight-dimensional process parameter space, the resulting sample size remained relatively limited for training multi-output ML models. To improve the diversity of the training data while preserving the experimentally established process–property relationships, a controlled perturbation-based data augmentation strategy was employed.

Synthetic observations were generated by randomly selecting observations from the original experimental dataset and applying stochastic perturbations to the continuous process parameters and their corresponding mechanical responses. For each continuous variable, the perturbation was generated from a zero-mean Gaussian distribution, with the standard deviation defined as 2% of the experimentally observed range of that variable. Thus, the 2% value represents the standard deviation of the Gaussian perturbation rather than a fixed ±2% change or a uniform perturbation. The perturbed values were subsequently constrained within the experimentally observed minimum–maximum range. The same perturbation procedure was applied to the corresponding mechanical-response variables to retain the local pairing between each process-parameter observation and its associated response vector. Raster angle and build orientation were retained unchanged because they represent categorical manufacturing settings. A fixed random seed of 42 was used to ensure reproducibility of the augmentation procedure. Using this procedure, 49 additional observations were generated, expanding the dataset from 51 experimental observations to 100 observations.

(1) $$X_{aug}=\mathrm{clip}\left(X+\epsilon ,X_{min},X_{max}\right),\;\;\;\;\epsilon ∼N\left(0,{\left[0.02\left(X_{max}-X_{min}\right)\right]}^{2}\right)$$

where X denotes the original value of a continuous process parameter or mechanical response, $X_{aug}$ denotes the corresponding augmented value, $X_{min}$ and $X_{max}$ are the experimentally observed minimum and maximum values of that variable, and ϵ represents the randomly generated perturbation. Here, N denotes the normal (Gaussian) distribution, with zero mean and a standard deviation equal to 2% of the observed range $\left(X_{max}-X_{min}\right)$. The clip operation constrains the augmented value within the experimentally observed minimum–maximum range.

To prevent information leakage and ensure an unbiased evaluation, data augmentation was performed independently within each training fold of the subsequent Leave-One-Out Cross-Validation (LOOCV) procedure, while the corresponding test observation remained entirely unseen during model training. The synthetic samples were intended solely to improve the diversity of the training data available to the learning algorithms and should not be regarded as a substitute for additional independent experimental observations. An ablation comparison between the original 51-observation dataset and the augmented 100-observation dataset is presented in Section 3.2.2 to evaluate the influence of the augmentation strategy on model performance.

Following augmentation, the predictor variables were standardized using the StandardScaler technique so that each feature had zero mean and unit variance according to

(2) $$z=\frac{x-\mu }{\sigma }$$

where *x* is the original feature value, *μ* is the mean of the corresponding feature, and *σ* is its standard deviation. Standardization improves numerical stability and prevents variables with larger numerical ranges from disproportionately influencing distance- and gradient-based learning algorithms.

Subsequently, five representative supervised ML regression algorithms—Random Forest (RF), Support Vector Regression (SVR), Artificial Neural Network (ANN), K-Nearest Neighbours (KNN), and Extreme Gradient Boosting (XGBoost)—were systematically benchmarked using the LOOCV strategy described in Section 2.4. To ensure a fair and reproducible comparison, all candidate models were trained using the same pre-processed dataset and evaluated under an identical validation protocol. Predictive performance was assessed using the coefficient of determination (R^2^) and the Root Mean Square Error (RMSE). SVR achieved the highest overall predictive performance (mean R^2^ = 0.9939, RMSE = 1.0159), followed closely by ANN (R^2^ = 0.9927, RMSE = 1.0983). Considering both their superior predictive performance and complementary learning mechanisms, these two heterogeneous algorithms were selected as the base learners for the proposed stacked learning framework.

To ensure a consistent and reproducible comparison, all candidate machine learning models were implemented using predefined hyperparameter settings. RF, SVR, ANN, KNN, and RR were implemented using the Scikit-learn library, whereas Extreme Gradient Boosting (XGBoost) was implemented using the XGBoost Python (3.2.0) package. The complete hyperparameter settings adopted for the candidate models and the RR meta-learner are summarized in Table 3.

**Figure 3:**
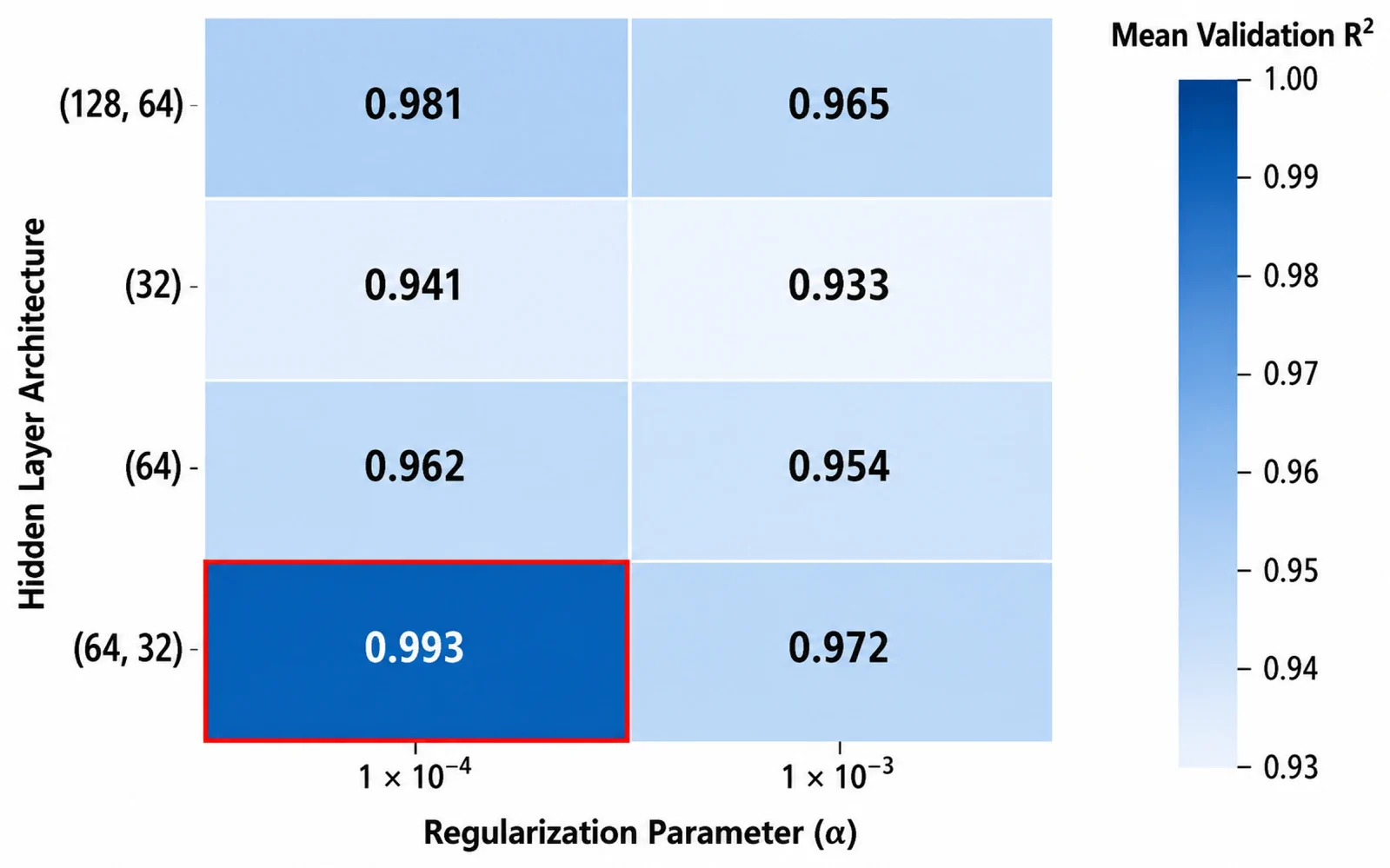
Heatmap of Grid Search results for ANN hyperparameter optimization. The optimal configuration (64, 32) with α = 1 × 10^−4^ achieved the highest mean validation R^2^ (0.993) and was used in the final model.

To select the ANN architecture, hyperparameter tuning was performed using Grid Search combined with five-fold cross-validation (Figure 3). Different hidden-layer configurations and L2 regularization coefficients (α) were systematically evaluated while maintaining the ReLU activation function and Adam optimizer. The search identified the architecture comprising two hidden layers of (64, 32) with an alpha value of 0.0001 as the best-performing configuration, yielding the highest average cross-validation score (R^2^ = 0.993). The resulting optimized ANN configuration was subsequently employed throughout all comparative analyses.

### 2.3. Proposed Heterogeneous Multi-Output Stacked Learning Framework

Following the candidate model screening presented in Section 2.2, Support Vector Regression (SVR) and an Artificial Neural Network (ANN) were selected as the heterogeneous base learners owing to their superior predictive performance. Although SVR achieved the highest overall prediction accuracy, ANN exhibited comparable performance while employing a fundamentally different nonlinear learning mechanism. Consequently, the two algorithms were expected to capture complementary characteristics of the complex process–property relationships. Stacked learning was therefore adopted to integrate these complementary prediction spaces, enabling the meta-learner to exploit the strengths of both models while reducing model-specific prediction errors. The proposed framework employs a two-level heterogeneous stacked learning architecture in which the prediction outputs of the two base learners are integrated through Ridge Regression (RR) to simultaneously predict the five mechanical properties of FDM-fabricated ASA components. Ridge Regression was selected as the meta-learner because its L2 regularization effectively mitigates overfitting while efficiently combining the correlated prediction outputs generated by the heterogeneous base learners. The overall architecture of the proposed framework is illustrated in Figure 1.

The supervised learning dataset is represented as

(3) $$D={\left(x_{i},y_{i}\right)}_{\left(i=1\right)}^{N}$$

where N denotes the total number of experimental observations, and $x_{i}$ represents the standardized input vector (ET, BT, ID, LH, PS, RA, BO, and CFS), whereas $y_{i}$ denotes the corresponding response vector (TS, FS, CS, RH, and CIS).

During the first stage of the proposed framework, the standardized input dataset was independently processed by the SVR and ANN base learners. Both heterogeneous learners received the same input feature matrix, ensuring that any differences in prediction arose solely from their distinct learning mechanisms rather than differences in the input information. Since SVR and ANN employ fundamentally different learning strategies, their prediction outputs capture complementary aspects of the underlying nonlinear process–property relationships, thereby providing richer predictive information for the subsequent meta-learning stage. The prediction process of the heterogeneous base learners is expressed as

(4) $${Ŷ}_{SVR}=f_{SVR}\left(X\right),\;{Ŷ}_{ANN}=f_{ANN}\left(X\right)$$

where ${Ŷ}_{SVR}$ and ${Ŷ}_{ANN}$ denote the multi-output prediction matrices generated by the SVR and ANN base learners, respectively. Each learner independently estimates all five mechanical properties from the same standardized process parameter matrix, thereby producing complementary prediction spaces.

The prediction matrices generated by the heterogeneous base learners were subsequently concatenated to construct the meta-feature matrix:

(5) $$Z=\left[{Ŷ}_{SVR},{Ŷ}_{ANN}\right]$$

where Z represents the combined prediction space that serves as the input to the RR meta-learner. This concatenated representation enables the meta-learner to identify the optimal combination of the complementary prediction spaces generated by SVR and ANN, thereby reducing individual model-specific prediction errors and improving overall predictive performance.

To ensure leakage-free meta-learning, the prediction matrices used to construct Z were generated using five-fold internal cross-validation within each outer LOOCV training fold. Specifically, the SVR and ANN base learners generated out-of-fold predictions for the training observations, and these predictions were concatenated to form the meta-feature matrix Z. The Ridge Regression meta-learner was then trained exclusively on these out-of-fold meta-features. The outer LOOCV test observation was excluded from the generation of the training meta-features and from the training of the meta-learner, thereby preventing information leakage between the base-learning and meta-learning stages.

After training the meta-learner, the SVR and ANN base learners were refitted using the complete augmented outer training set and used to generate predictions for the held-out outer test observation, which was then passed to the trained Ridge Regression meta-learner to obtain the final prediction.

Accordingly, the overall prediction process of the proposed heterogeneous stacked learning framework can be summarized as

(6) $$Ŷ=f_{RR}\left(f_{SVR}\left(X\right),f_{ANN}\left(X\right)\right)$$

where Ŷ denotes the final multi-output prediction matrix comprising TS, FS, CS, RH, and CIS.

By hierarchically integrating the kernel-based regression capability of SVR with the nonlinear feature-learning capability of ANN through a regularized Ridge Regression meta-learning stage, the proposed framework combines complementary predictive information to generate more robust and reliable simultaneous predictions of TS, FS, CS, RH, and CIS than either standalone learner.

The overall computational workflow of the proposed heterogeneous stacked learning framework can be summarized as follows: (i) the standardized input features are independently processed by the SVR and ANN base learners to generate multi-output predictions; (ii) the prediction outputs of both learners are concatenated to form the meta-feature matrix; and (iii) RR combines these complementary prediction spaces to generate the final multi-output predictions. This hierarchical learning strategy enables the framework to exploit the strengths of heterogeneous learning algorithms while improving prediction robustness and generalization.

### 2.4. Model Validation and Performance Evaluation

To obtain a rigorous and unbiased assessment of predictive performance, all candidate machine learning models (SVR, ANN, RF, KNN, and XGBoost), together with the proposed heterogeneous stacked learning framework, were evaluated using the Leave-One-Out Cross-Validation (LOOCV) strategy. The experimental dataset comprised 17 unique process parameter combinations generated through the Definitive Screening Design (DSD), with each condition fabricated and mechanically tested in triplicate, yielding 51 experimental observations. Following the augmentation strategy described in Section 2.2, the training dataset within each LOOCV iteration was expanded to improve sample diversity, whereas the corresponding test observation remained entirely independent and was never augmented. Consequently, each observation served exactly once as the test sample, while all remaining observations constituted the training data, providing a consistent evaluation of predictive performance while preventing information leakage between training and testing.

LOOCV was selected because it maximizes the use of the experimentally generated dataset while providing an almost unbiased estimate of predictive performance for relatively small datasets. Given the limited number of experimental observations available in this study, reserving larger hold-out subsets would substantially reduce the amount of information available for model training. Although the dataset comprises replicated measurements from 17 unique processing conditions, the adopted validation strategy provides a consistent basis for comparing all candidate models under identical conditions. Future work will investigate validation using larger independently generated datasets and group-based validation strategies to further assess model generalizability across unseen processing conditions.

The predictive performance of all candidate machine learning models and the proposed heterogeneous stacked learning framework was evaluated using the coefficient of determination (R^2^) and the root mean square error (RMSE). The coefficient of determination quantifies the proportion of variance in the experimental data explained by the predictive model, whereas RMSE measures the average magnitude of prediction error while assigning greater weight to larger deviations. The simultaneous use of these complementary metrics provides a balanced assessment of both predictive accuracy and error magnitude.

The coefficient of determination is expressed as

(7) $$R^{2}=1-\frac{\sum_{i=1}^{N}(y_{i}-{\hat{y}}_{i}{)}^{2}}{\sum_{i=1}^{N}(y_{i}-\bar{y}{)}^{2}}$$

where $y_{i}$, ${\hat{y}}_{i}$, and $\bar{y}$ denote the experimentally measured value, predicted value, and mean of the experimental observations, respectively.

The RMSE is calculated as

(8) $$RMSE=\sqrt{\frac{1}{N}\sum_{i=1}^{N}(y_{i}-{\hat{y}}_{i}{)}^{2}}$$

where N represents the total number of observations.

Models exhibiting higher R^2^ values together with lower RMSE values were considered to demonstrate superior predictive performance. The identical validation protocol and evaluation metrics were applied consistently to all candidate models and the proposed heterogeneous stacked learning framework to ensure a fair, reproducible, and unbiased comparison of their predictive capabilities.

## 3. Results and Discussion

### 3.1. Benchmarking of Candidate Machine Learning Models

To identify the most suitable base learners for the proposed heterogeneous stacked learning framework, five supervised machine learning algorithms—Support Vector Regression (SVR), Artificial Neural Network (ANN), Random Forest (RF), K-Nearest Neighbours (KNN), and Extreme Gradient Boosting (XGBoost)—were systematically benchmarked using the validation strategy described in Section 2.4. Their predictive performance was evaluated using the mean coefficient of determination ($R^{2}$) and root mean square error (RMSE), as summarized in Table 4.

The comparative results reveal that SVR achieved the highest predictive accuracy, attaining a mean $R^{2}$ of 0.9939 with the lowest RMSE (1.0159), indicating its strong capability to model the nonlinear relationships between the FDM process parameters and the multiple mechanical properties of ASA. ANN exhibited comparable performance, with a mean $R^{2}$ of 0.9927 and an RMSE of 1.0983, confirming its effectiveness in learning complex nonlinear input–output relationships from the experimental dataset. Although RF and KNN also demonstrated high predictive accuracy, their slightly lower $R^{2}$ values (0.9890 and 0.9863, respectively) and comparatively higher RMSE values suggest a modest reduction in prediction precision. Among the evaluated models, XGBoost exhibited the lowest predictive performance, yielding a mean $R^{2}$ of 0.965 and the highest RMSE (2.4468).

Based on their superior predictive performance, SVR and ANN were selected as the heterogeneous base learners for the proposed stacked learning framework, with Ridge Regression used as the meta-learner.

The reported R^2^ and RMSE values represent cross-validated predictive performance obtained through the LOOCV procedure described in Section 2.4. The differences among SVR, ANN, RF, and KNN were relatively small, whereas XGBoost exhibited comparatively lower predictive accuracy. The influence of data augmentation on predictive performance is further examined through the comparison of the original and augmented datasets presented in Section 3.2.2.

### 3.2. Comparative Performance Evaluation of the ML Models

#### 3.2.1. Comparative Prediction Analysis

Following the screening results in Section 3.1, the predictive performance of SVR, ANN, and the proposed hybrid framework was compared under the same LOOCV protocol.

As summarized in Table 5, all three models achieved overall mean R^2^ values above 0.99. The proposed hybrid framework attained the highest overall R^2^ (0.9943) and lowest RMSE (0.9758), followed by SVR (R^2^ = 0.9939; RMSE = 1.0159) and ANN (R^2^ = 0.9927; RMSE = 1.0983). Although the numerical improvements over the standalone models were modest, the hybrid framework consistently achieved the highest R^2^ across all five mechanical properties. In this high-performance regime, even small increases in R^2^ accompanied by reductions in RMSE indicate that the stacked framework successfully exploited complementary predictive information that was not fully captured by either base learner individually.

**Figure 4:**
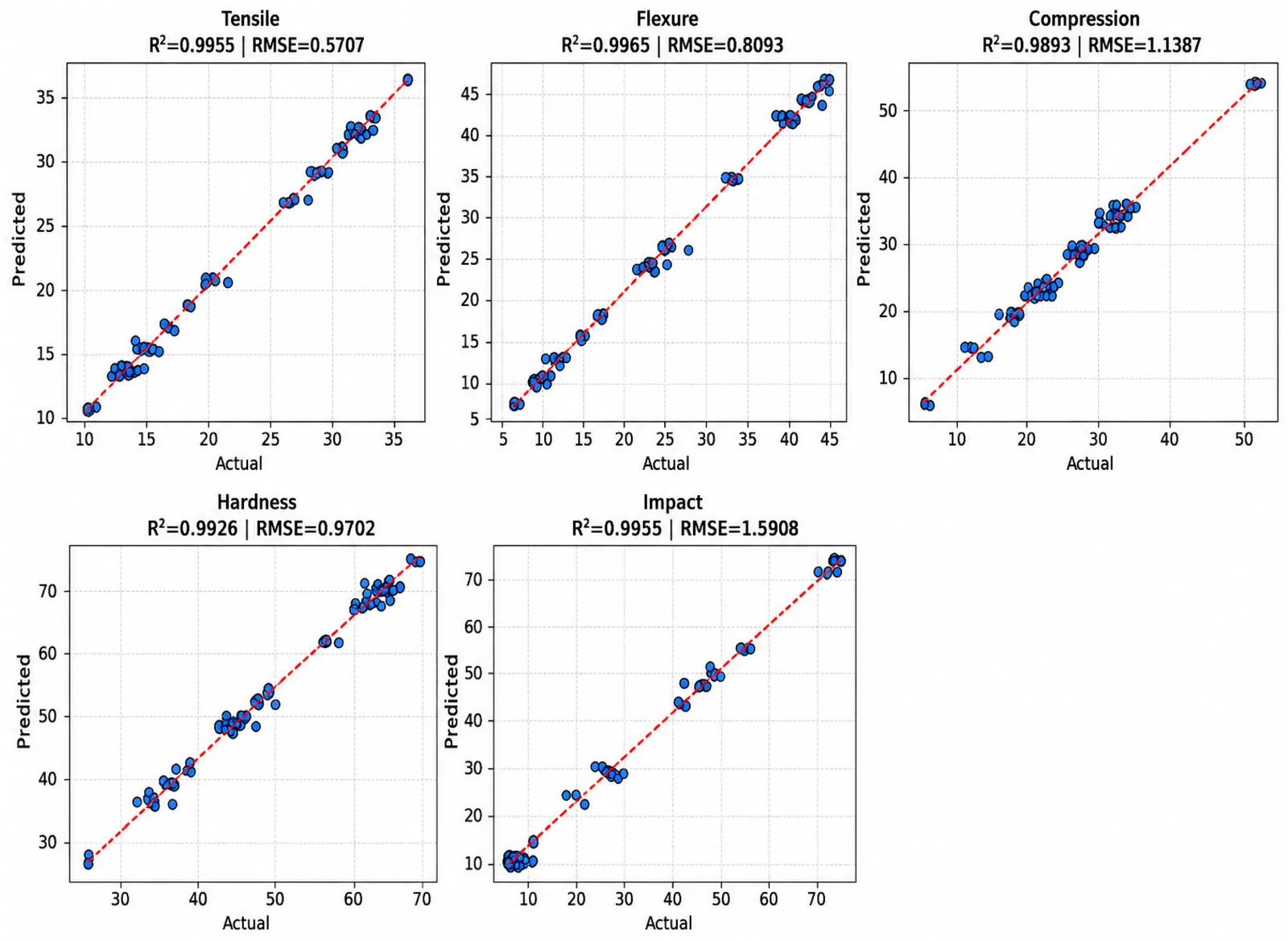
Prediction parity plots comparing the experimental and predicted values of the five mechanical properties obtained using the SVR model.

**Figure 5:**
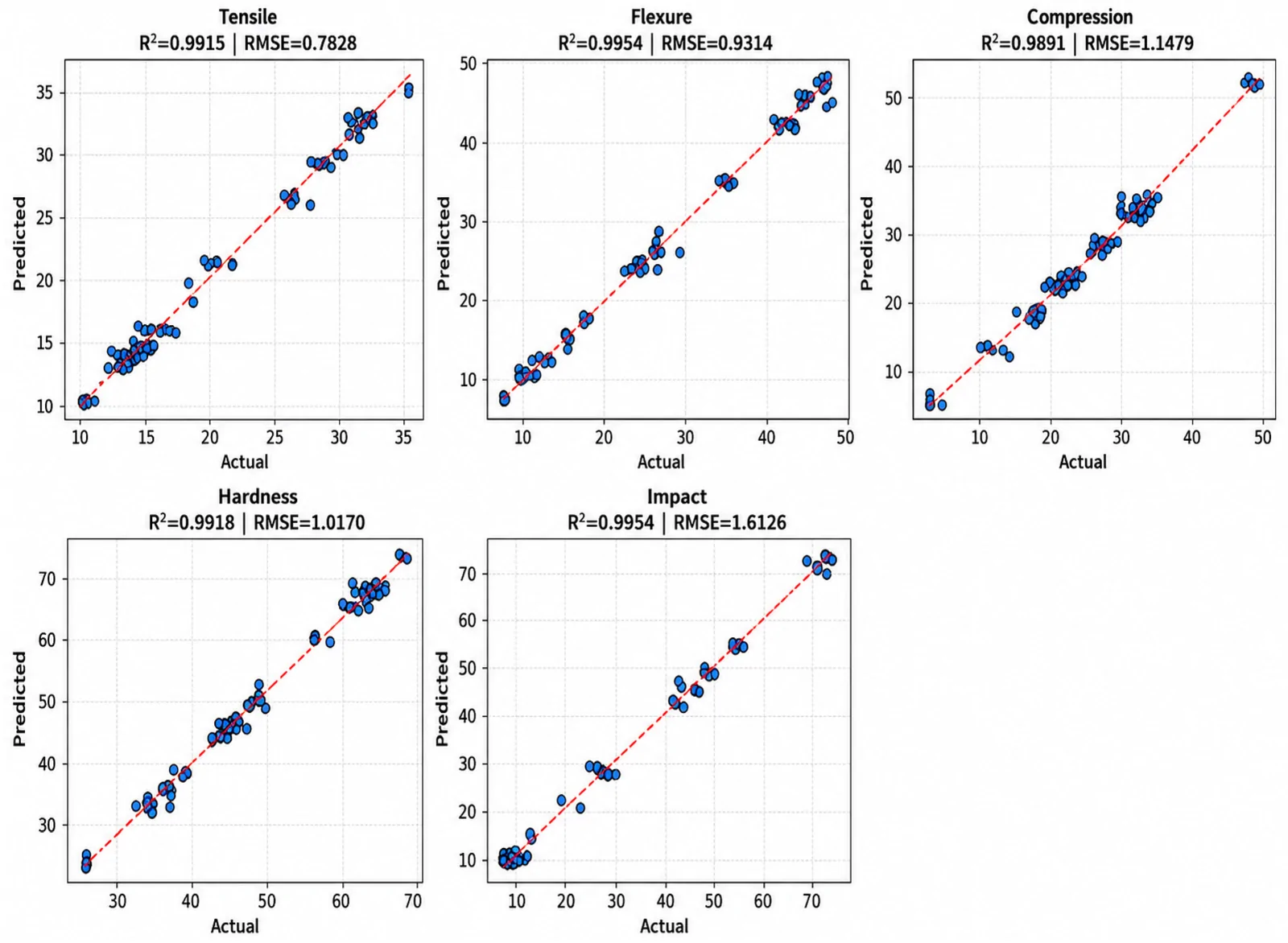
Prediction parity plots comparing the experimental and predicted values of the five mechanical properties obtained using the ANN model.

**Figure 6:**
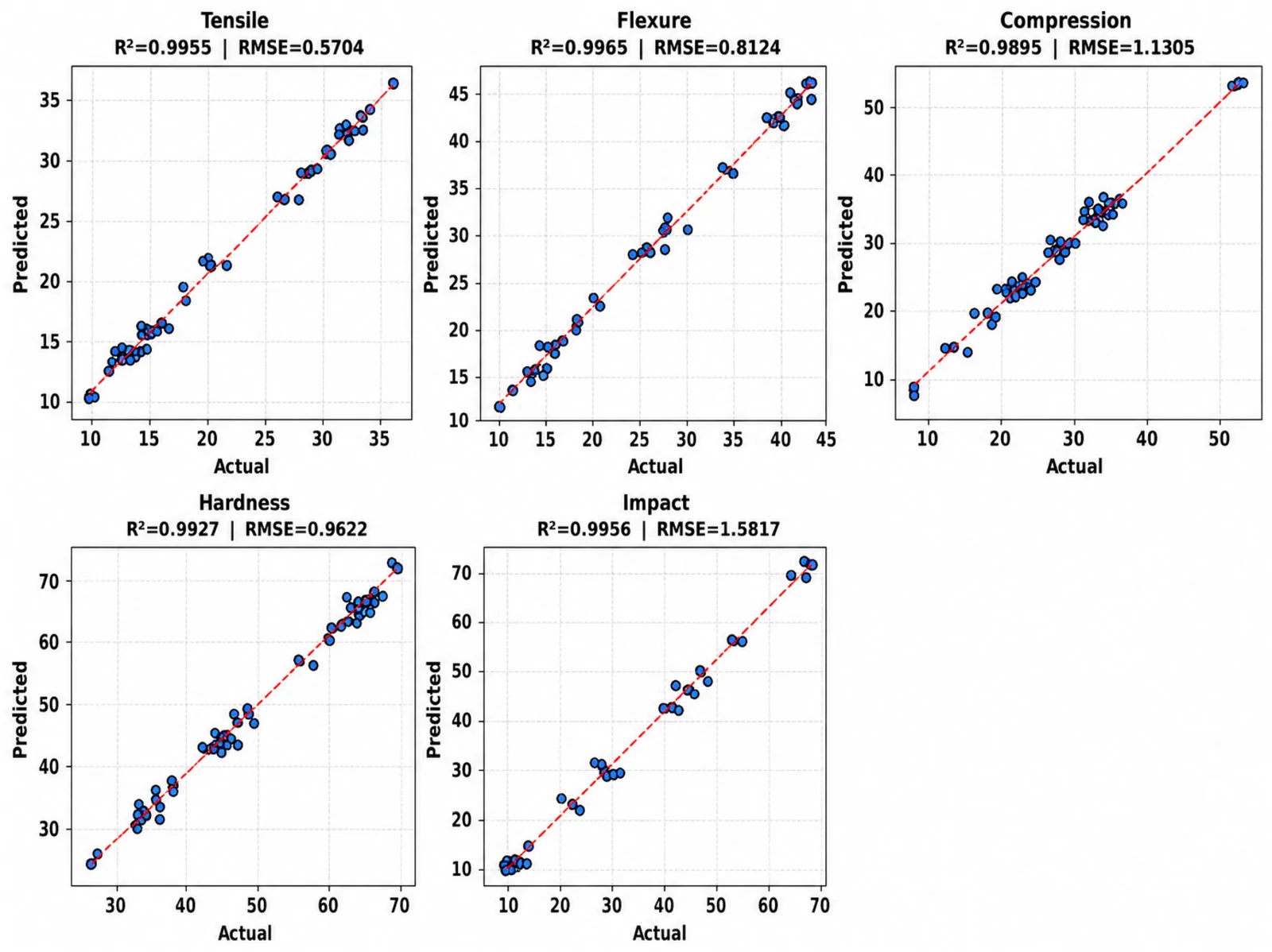
Prediction parity plots comparing the experimental and predicted values of the five mechanical properties obtained using the proposed heterogeneous stacked learning framework.

The consistent improvement across the five responses indicates that the proposed framework provides a modest but consistent gain over the standalone base learners under the same validation protocol. The developed models establish predictive relationships between the eight investigated FDM process parameters and the five mechanical responses within the investigated experimental domain. Detailed experimental–predicted parity plots (Figure 4, Figure 5 and Figure 6), together with the property-specific performance metrics reported in Table 5, demonstrate the predictive capability of the developed models.

#### 3.2.2. Effect of Data Augmentation

To evaluate the influence of data augmentation, an ablation study was conducted comparing the original 51 experimental observations with the augmented 100-observation dataset under the same validation framework. The results are presented in Table 6.

The augmentation strategy substantially improved the predictive performance of all three models. For SVR, R^2^ increased from 0.8977 to 0.9939, while RMSE decreased from 3.3875 to 1.0159, corresponding to a 70.01% reduction in RMSE. ANN exhibited an increase in R^2^ from 0.7958 to 0.9927, accompanied by a reduction in RMSE from 5.4392 to 1.0983, representing a 79.81% reduction. The proposed heterogeneous stacked framework also showed a marked improvement, with R^2^ increasing from 0.8244 to 0.9943 and RMSE decreasing from 4.8922 to 0.9758, corresponding to an 80.05% reduction in RMSE. These results demonstrate that the controlled augmentation strategy substantially enhanced predictive performance under the adopted validation framework. However, the augmented observations represent synthetic data enrichment and should not be interpreted as additional independent experimental observations.

#### 3.2.3. Prediction Agreement and Error Analysis

**Figure 7:**
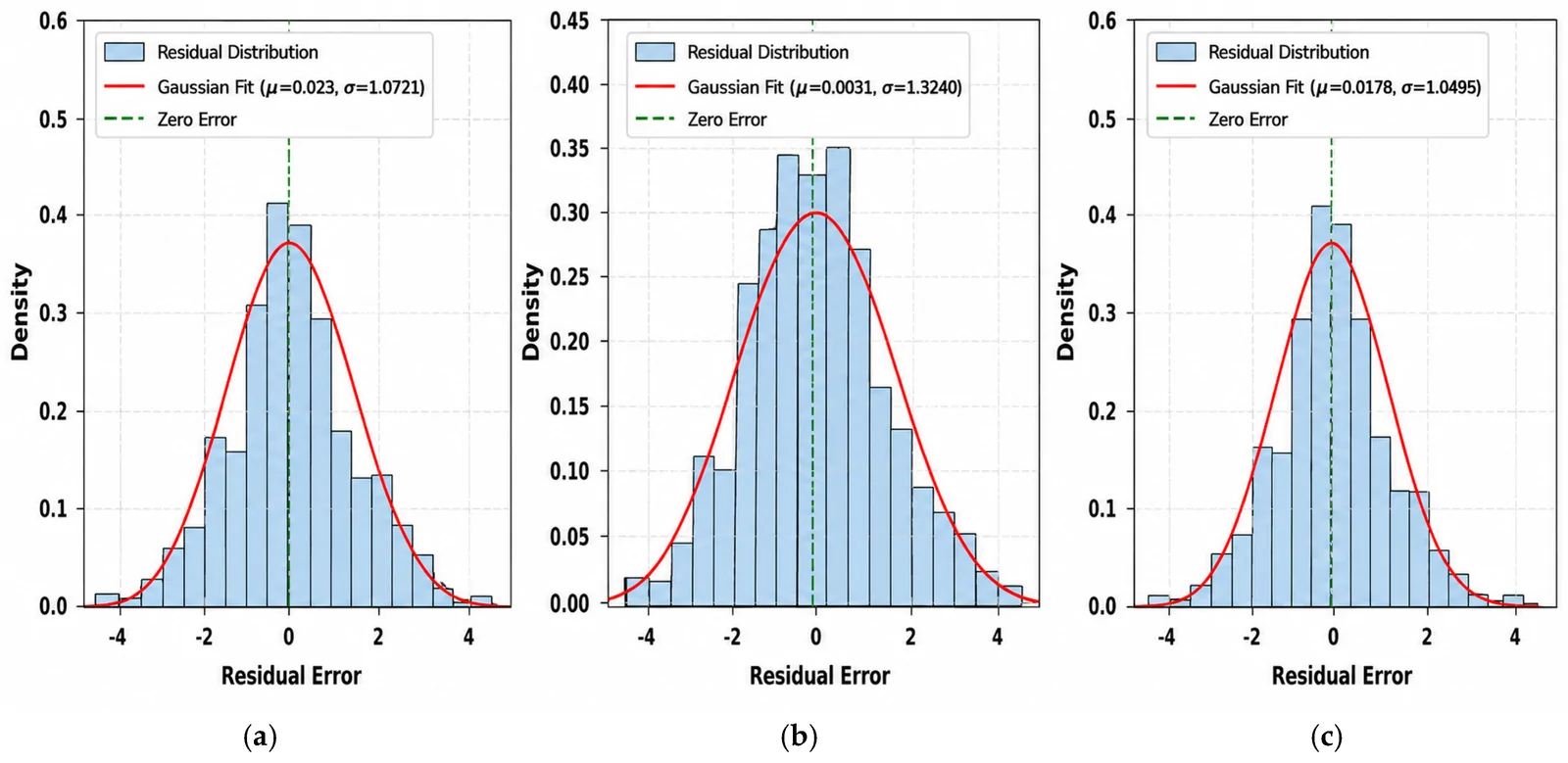
Residual error distributions of the (**a**) SVR, (**b**) ANN, and (**c**) hybrid models under LOOCV. The red curves represent Gaussian fits, and the green dashed lines indicate zero prediction error. The hybrid model exhibits the lowest residual dispersion (σ = 1.0667).

While Table 5 quantifies the superior overall performance of the proposed heterogeneous stacked learning framework, the parity plots (Figure 4, Figure 5 and Figure 6) and residual distributions (Figure 7) provide deeper insight into prediction behaviour across the response domain. These graphical analyses complement the statistical metrics by revealing prediction consistency, error dispersion, and robustness that cannot be inferred from the coefficient of determination (R^2^) and root mean square error (RMSE) alone.

All three models successfully capture the nonlinear relationships between the FDM process parameters and the five mechanical properties of ASA, with predicted values closely distributed around the identity line. However, clear differences in prediction compactness are evident. Compared with ANN (Figure 5), the SVR model (Figure 4) produces a more concentrated prediction distribution, consistent with its slightly higher predictive performance reported in Table 5, suggesting that the kernel-based regression strategy provides marginally better generalization for the present experimental dataset. Among the investigated responses, CS exhibits the largest prediction scatter in both models, indicating that it is the most challenging mechanical property to predict.

The hybrid model (Figure 6) further contracts the prediction cloud toward the identity line without altering the overall nonlinear trend established by the base learners. The improvement is modest for TS and FS, where the standalone models already exhibit excellent agreement with the experimental observations, but becomes more apparent for CS, RH, and CIS, which display comparatively greater prediction dispersion in the individual models. These results indicate that heterogeneous stacking is particularly beneficial when the process–property relationship is difficult to approximate with a single learning algorithm, thereby enhancing the consistency of the overall multi-output prediction task.

The residual distributions reinforce these observations. All three models yield approximately Gaussian residuals centred close to zero, confirming the absence of appreciable systematic prediction bias. The hybrid framework produces the narrowest residual distribution (σ = 1.0667), followed by SVR (σ = 1.0721) and ANN (σ = 1.1343). Although the reduction in residual dispersion is modest, it occurs concurrently with improvements in both the overall R^2^ and RMSE and is consistently maintained across the complete multi-output prediction problem. Furthermore, the consistent reduction in prediction dispersion under the LOOCV validation strategy indicates that the improved performance reflects enhanced predictive robustness rather than favourable data partitioning.

Notably, the hybrid framework enhances the overall quality and consistency of prediction while preserving excellent predictive accuracy across all five mechanical properties. It reduces local prediction deviations for the majority of the investigated responses without compromising the strong predictive capability already achieved by the standalone models. Such balanced behaviour is particularly valuable for multi-output modelling in additive manufacturing, where multiple performance indicators must be estimated simultaneously from a common set of process variables.

To complement the pooled residual distributions shown in Figure 7, the property-specific residual standard deviations are reported in their original engineering units in Table 7. This representation allows the magnitude of the prediction error to be interpreted directly for each mechanical property.

The property-specific residual dispersion further demonstrates the relative prediction accuracy in physically interpretable units. The hybrid model exhibits the lowest residual standard deviation for all five mechanical properties, with σ values of 2.7161 HRM for RH, 4.4206 kJ/m^2^ for CIS, 2.4367 MPa for CS, 3.7996 MPa for FS, and 1.3628 MPa for TS. These values provide a direct measure of prediction dispersion in the natural units of each response and complement the pooled residual statistics presented in Figure 7. The consistently lower dispersion of the hybrid model across all responses supports its improved prediction consistency under the grouped LOOCV framework.

## 4. Conclusions

This study developed and experimentally validated a heterogeneous multi-output stacked learning framework for the simultaneous prediction of the mechanical properties of FDM-fabricated ASA. Using a Definitive Screening Design (DSD)-based dataset comprising 51 experiments (augmented to 100), eight FDM process parameters, and five mechanical responses, five supervised learning algorithms were benchmarked to identify the optimal base learners. The proposed hybrid framework, integrating SVR and ANN through Ridge Regression, achieved the highest overall predictive performance (R^2^ = 0.9943, RMSE = 0.9758), outperforming the standalone SVR (R^2^ = 0.9939, RMSE = 1.0159) and ANN (R^2^ = 0.9927, RMSE = 1.0983) models while exhibiting the lowest residual dispersion (σ = 1.0667).

The principal contribution of this work is the establishment of an experimentally validated heterogeneous multi-output prediction framework that effectively exploits the complementary learning characteristics of SVR and ANN to achieve reliable simultaneous prediction of multiple mechanically distinct properties within a unified model. Beyond improving predictive performance, the proposed AI-assisted methodology provides a practical framework for supporting data-driven analysis, intelligent process optimization, and enhanced understanding of process–structure–property relationships in FDM-fabricated engineering polymers. Consequently, the proposed framework contributes to the advancement of intelligent polymer additive manufacturing by enabling more reliable predictive decision-making and reducing reliance on extensive experimental trial-and-error during process development. Future work will focus on validating the proposed framework using larger independent experimental datasets, extending its applicability to additional engineering polymers and additive manufacturing processes, and integrating explainable artificial intelligence with multi-objective optimization to further enhance model interpretability, generalizability, and intelligent manufacturing decision-making.

## Figures and Tables

**Table 1:** Process parameters and their levels.

Process Parameter	Symbol	Level		
		−1	0	+1
Extruder temperature (°C)	ET	240	250	260
Bed temperature (°C)	BT	75	85	95
Infill density (%)	ID	50	65	80
Layer height (mm)	LH	0.1	0.2	0.3
Print speed (mm/s)	PS	35	50	65
Raster angle (°)	RA	0	45	90
Build orientation (°)	BO	0	45	90
Cooling fan speed (%)	CFS	20	35	50

**Table 2:** Definitive screening design for experimental runs.

Exp. Run	ET	BT	ID	LH	PS	RA	BO	CFS
1	250	95	80	0.3	65	90	90	50
2	250	75	50	0.1	35	0	0	20
3	260	85	80	0.1	65	0	0	50
4	240	85	50	0.3	35	90	90	20
5	260	75	65	0.1	65	90	90	20
6	240	95	65	0.3	35	0	0	50
7	260	95	80	0.2	35	90	0	20
8	240	75	50	0.2	65	0	90	50
9	260	75	50	0.3	50	90	0	50
10	240	95	80	0.1	50	0	90	20
11	260	95	50	0.1	35	45	90	50
12	240	75	80	0.3	65	45	0	20
13	260	95	50	0.3	65	0	45	20
14	240	75	80	0.1	35	90	45	50
15	260	75	80	0.3	35	0	90	35
16	240	95	50	0.1	65	90	0	35
17	250	85	65	0.2	50	45	45	35

**Table 3:** Hyperparameter settings of the candidate machine learning models and the Ridge Regression meta-learner.

Model	Hyperparameter Settings
**SVR**	Kernel = RBF; C = 100; γ = scale; ε = 0.1
**ANN (MLPRegressor)**	ANN (MLPRegressor)|Hidden layers = (64, 32); Activation = ReLU; Optimizer = Adam; Learning rate = 0.001; Alpha = 0.0001; Max iterations = 2000; Random state = 42. Hyperparameters were selected using Grid Search with five-fold cross-validation.
**Random Forest**	n_estimators = 300; max_depth = 5; min_samples_split = 8; min_samples_leaf = 4; max_features = √(total features); bootstrap = True
**KNN**	n_neighbors = 7; weights = distance; metric = Euclidean; algorithm = brute
**XGBoost**	learning_rate = 0.05; max_depth = 3; n_estimators = 150; min_child_weight = 5; subsample = 0.8; colsample_bytree = 0.8; gamma = 1; reg_alpha = 0.5; reg_lambda = 1
**Ridge Regression** **(Meta-learner)**	α = 1.0

**Table 4:** Comparative overall performance of candidate ML models during the screening stage.

Model	Mean R^2^	RMSE
Support Vector Regression (SVR)	0.9939	1.0159
Artificial Neural Network (ANN)	0.9927	1.0983
Random Forest (RF)	0.9890	1.3660
K-Nearest Neighbours (KNN)	0.9863	1.4132
Extreme Gradient Boosting (XGBoost)	0.9652	2.4468

**Table 5:** Comparative predictive performance of the ML models.

Mechanical Property	SVR (R^2^)	ANN (R^2^)	Proposed Framework (R^2^)
Tensile Strength (TS)	0.9955	0.9915	0.9957
Flexural Strength (FS)	0.9965	0.9954	0.9967
Compression Strength (CS)	0.9893	0.9891	0.9898
Rockwell Hardness (RH)	0.9926	0.9918	0.9929
Charpy Impact Strength (CIS)	0.9955	0.9954	0.9964
Overall Mean R^2^	0.9939	0.9927	0.9943
RMSE	1.0159	1.0983	0.9758

**Table 6:** Ablation comparison of model performance using the original and augmented datasets.

Performance Metric	SVR	ANN	Proposed Framework
R^2^ (51 records)	0.8977	0.7958	0.8244
R^2^ (100 records)	0.9939	0.9927	0.9943
ΔR^2^	0.0962	0.1969	0.1699
RMSE (51 records)	3.3875	5.4392	4.8922
RMSE (100 records)	1.0159	1.0983	0.9758
RMSE reduction (%)	70.01	79.81	80.05

**Table 7:** Property-specific residual standard deviation (σ) of the ML models in the original units of each mechanical property.

Model	σ_RH_ (HRM)	σ_CIS_ (kJ/m^2^)	σ_CS_ (MPa)	σ_FS_ (MPa)	σ_TS_ (MPa)
**SVR**	3.4511	4.5834	2.6883	3.8531	1.3853
**ANN**	5.0404	7.9314	4.9107	5.9101	2.3154
**Hybrid**	2.7161	4.4206	2.4367	3.7996	1.3628

## Data Availability

The original contributions presented in this study are included in the article. Further inquiries can be directed to the corresponding author.

## Abbreviations

The following abbreviations are used in this manuscript:

ANN	Artificial Neural Network
ASA	Acrylonitrile Styrene Acrylate
BO	Build Orientation
BT	Bed Temperature
CFS	Cooling Fan Speed
CIS	Charpy Impact Strength
CS	Compressive Strength
DSD	Definitive Screening Design
ET	Extruder Temperature
FDM	Fused Deposition Modeling
FS	Flexural Strength
ID	Infill Density
ML	Machine Learning
PS	Print Speed
RA	Raster Angle
RF	Random Forest
RH	Rockwell Hardness
RMSE	Root Mean Square Error
RR	Ridge Regression
SVR	Support Vector Regression
TS	Tensile Strength
XGBoost	Extreme Gradient Boosting
LH	Layer Height
KNN	K-Nearest Neighbours
